# Selection of marker genes for genetic barcoding of microorganisms and binning of metagenomic reads by Barcoder software tools

**DOI:** 10.1186/s12859-018-2320-1

**Published:** 2018-08-30

**Authors:** Adeola M. Rotimi, Rian Pierneef, Oleg N. Reva

**Affiliations:** 1 Centre for Bioinformatics and Computational Biology, Dep. Biochemistry, University of Pretoria, Lynnwood Rd, Hillcrest, Pretoria, 0002 South Africa; 2 Biotechnology Platform, Agricultural Research Council, Onderstepoort, South Africa

**Keywords:** Metabarcoding, Metagenome, NGS, Bacterial genome, Software tool

## Abstract

**Background:**

Metagenomic approaches have revealed the complexity of environmental microbiomes with the advancement in whole genome sequencing displaying a significant level of genetic heterogeneity on the species level. It has become apparent that patterns of superior bioactivity of bacteria applicable in biotechnology as well as the enhanced virulence of pathogens often requires distinguishing between closely related species or sub-species. Current methods for binning of metagenomic reads usually do not allow for identification below the genus level and generally stops at the family level.

**Results:**

In this work, an attempt was made to improve metagenomic binning resolution by creating genome specific barcodes based on the core and accessory genomes. This protocol was implemented in novel software tools available for use and download from http://bargene.bi.up.ac.za/. The most abundant barcode genes from the core genomes were found to encode for ribosomal proteins, certain central metabolic genes and ABC transporters. Performance of metabarcode sequences created by this package was evaluated using artificially generated and publically available metagenomic datasets. Furthermore, a program (Barcoding 2.0) was developed to align reads against barcode sequences and thereafter calculate various parameters to score the alignments and the individual barcodes. Taxonomic units were identified in metagenomic samples by comparison of the calculated barcode scores to set cut-off values. In this study, it was found that varying sample sizes, i.e. number of reads in a metagenome and metabarcode lengths, had no significant effect on the sensitivity and specificity of the algorithm. Receiver operating characteristics (ROC) curves were calculated for different taxonomic groups based on the results of identification of the corresponding genomes in artificial metagenomic datasets. The reliability of distinguishing between species of the same genus or family by the program was nearly perfect.

**Conclusions:**

The results showed that the novel online tool BarcodeGenerator (http://bargene.bi.up.ac.za/) is an efficient approach for generating barcode sequences from a set of complete genomes provided by users. Another program, Barcoder 2.0 is available from the same resource to enable an efficient and practical use of metabarcodes for visualization of the distribution of organisms of interest in environmental and clinical samples.

## Background

Metagenomics can be defined as a collection of techniques used for the direct investigation of genomes which contribute to an environmental or composite sample [1, 2]. Over the years, the field of metagenomics has transformed from sequencing of cloned DNA fragments using Sanger technology to direct sequencing (shotgun sequencing) of DNA without heterologous cloning [3–5]. Metagenomics offers: (i) access to the functional gene composition of microbial communities which enables a wider depiction than phylogenetic surveys and (ii) a strong tool for creating new hypotheses of microbial functions, e.g. the discovery of proteorhodopsin [4, 6].

Advances in sequencing technologies have provided researchers with the ability to promptly describe the microbial composition of an environmental or clinical sample with exceptional resolution. A wealth of genetic data has become available due to these approaches providing new understanding into environmental and human microbial ecology [7]. The reduction in the cost of sequencing has also rapidly enhanced the development of sequencing-based metagenomics. The number of metagenome shotgun sequence datasets has dramatically increased in the past few years [2]. Hence, metagenomic researchers have to analyse huge short-read datasets using tools designed for long-reads and more specifically for clonal datasets [5]. Binning is generally referred to as a method used for grouping reads or contigs and assigning them to operational taxonomic unit (OTUs). Various algorithms have been developed which make use of information contained within the given sequences. However, most of the methods used for binning of metagenomic reads do not allow for identification below the genus level and generally stop on the level of bacterial families [2].

Kress and Erickson (2008) defined DNA barcoding as a fast technique used for species identification based on nucleotide sequences [8]. However, since the single gene technique of DNA barcoding does not differentiate between closely related species and subspecies, it is of limited importance to develop markers in biotechnological and medical microbiology [9–11]. Hence, it was hypothesized that the comparison of bacterial strains by using multiple gene sequences would give a better resolution of their core relationship than a single gene [12]. The multilocus sequence typing (MLST) technique was introduced, which made use of DNA sequences of internal fragments of multiple housekeeping genes for a definitive identification of microorganisms [10, 13]. Various researchers have developed different techniques for MLST, some of which include ribosomal multilocus sequence typing (rMLST), multilocus sequence analysis (MLSA) and whole genome multilocus sequence typing (wgMLST) [14, 15]. The rMLST technique indexes variations seen in 53 genes encoding bacterial ribosome protein subunits (*rps* genes) as a way of incorporating microbial genealogy and typing. Groupings provided by rMLST were consistent with the present nomenclature systems independently of the clustering algorithm been used [14]. The MLSA technique is used to obtain a more advanced and better resolution of phylogenetic relationships of species within a genus. Partial sequences of genes coding for housekeeping genes are used to create phylogenetic trees and later to infer phylogenies in MLSA research. The MLSA technique has also been suggested as a replacement for DNA-DNA hybridization (DDH) in species delineation [15]. The two basic techniques used to create phylogenies for whole genome sequencing of enhanced outbreak surveys are: whole genome multilocus typing (wgMLST) and single nucleotide polymorphisms (SNPs). As with the traditional MLST, alleles in wgMLST are either the same or different, which implies that any nucleotide substitution, insertion or deletion is equivalent to one allele change. In wgMLST, several thousand loci can be matched. The estimated distances between them are then used to infer phylogenetic relationships by the clustering algorithms. For the SNP technique, changes seen in single nucleotide substitutions are used to deduce phylogenetic relatedness or genetic typing. The SNP protocol has been implemented in various software packages [16].

MLST approaches were promoted by the advances in next-generation sequencing (NGS). Different software applications have been developed using various techniques to calculate the sequence types (STs) from the NGS data. However, not all MLST calling applications are reliable. Challenges encountered with these programs include (i) computationally inefficient methods; (ii) false positive results; (iii) obsolescence of databases; (iv) inability to call alleles with low coverages; and (v) variable performances of mixed samples. Hence, there is room for improvement [16].

The aim of this study was to create an interactive computational service for the identification of the most suitable marker sequences for DNA-based multilocus barcoding. The basic idea was that the suitability of different marker genes would depend on the level of taxonomic relatedness between organisms to be distinguished or identified in environmental samples. In other words, marker genes selected to barcode organisms on the family or genus level most likely will not be suited to distinguish between species or subspecies. The program BarcoderGenerator, which is available online at http://bargene.bi.up.ac.za, creates genome specific barcodes based on the core and accessory genes from genome sequences provided by users. The proportion of accessory genes required can be selected alongside the desired length needed for the barcode sequences to be created. Another command-line application (Barcoder 2.0), available for download from the same web-interface, performs binning of metagenomics reads against generated barcodes and visualizes the results. It should be noted that these software tools were developed exclusively for metabarcoding, i.e. for identification of strains and species of interest in environmental samples by binning of metagenomics reads, and not for phylogenetic inferences. However, Barcoder 2.0 allows aligning of identified organisms along phylogenetic trees generated by other programs and saved in PHYLIP/Newick format.

## Implementation

For this study, different microorganisms were used in case studies: the *Escherichia* and *Shigella* group (40 strains), *Latobacillus* (30 strains), *Mycobacteria* (16 strains) and *Shewanella* (21 strains). Phylogenetic relationships between organisms of these groups were inferred by the alignment-free program SWPhylo available at http://swphylo.bi.up.ac.za/ [17]. The strains used represent different species and subspecies including pathogenic and biotechnological strains. Metagenomic datasets representing different eco-niches were obtained from the NCBI and MG-RAST databases [18]. Information about all bacterial genomes and metagenomic datasets used in this study, including resulting barcode sequences, are available from the help page (http://seqword.bi.up.ac.za/barcoder_help_download/).

The basic principles for selection of barcode sequences were detailed in a previous publication [11] and further developed in this work. The main idea was to identify clusters of orthologous genes (COGs) followed by codon alignment of COG sequences with the aim of identifying genes sufficiently conserved for proper identification and under positive selection of mutations to allow for distinguishing between organisms of interest. Statistical parameters used for scoring of marker sequences and the program outputs will be discussed in detail below.

For evaluation of the designed algorithm, Metasim [19] was used to generate collections of artificial reads simulating metagenome data sets. Sequence alignment was performed by MUSCLE [21]. Orthology prediction was done by reciprocal BLASTP implemented in an in-house Python script. For data visualization, *matplotlib* 1.5.1 (https://matplotlib.org/1.5.1/index.html) was used. All programs originating from this work were made accessible at http://bargene.bi.up.ac.za/ for download to be used with Python 2.7 (also compatible with Python 2.5).

## Results and discussion

### Selection of core genes for multilocus barcoding

Variable DNA sequences and protein molecules can be useful phylogenetic and taxonomic markers. While phylogenetics aims at inferring relationships of common ancestry, the objective of molecular barcoding is the identification of presence or absence of taxonomic units of interest in selected environmental samples or habitats. One classical example of bacterial barcode sequences is 16S rRNA – a sufficiently conserved gene, which can easily be identified in DNA reads and properly aligned. The barcode sequences should be variable enough to allow for a reliable identification of taxonomic units, but also have to be sufficiently conserved to avoid misalignments. Depending on the diversity of bacterial species to be distinguished, different genes may be better suited for the barcoding of organisms. Indeed, more versatile sequences will work for distinguishing between closely related species, while more conserved genes will be applicable for identification on higher taxonomic levels.

**Fig. 1:**
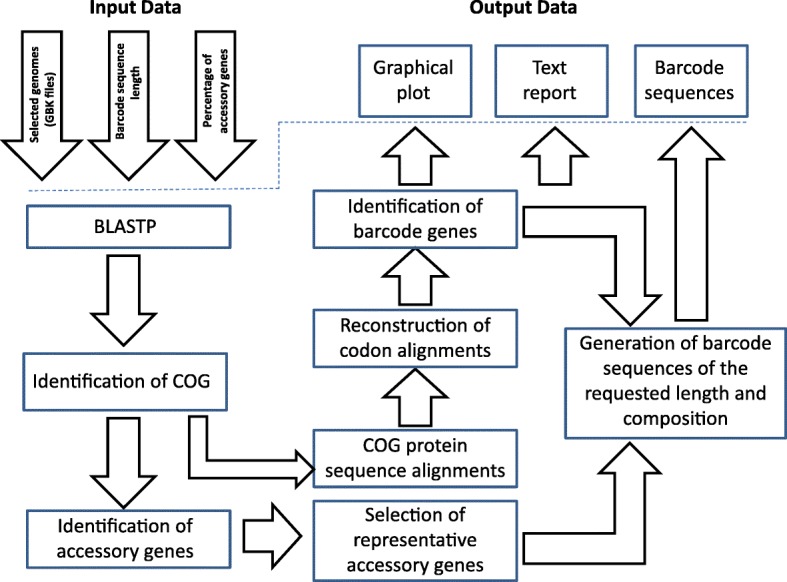
Workflow diagram of selection of diagnostic barcode sequences

**Fig. 2:**
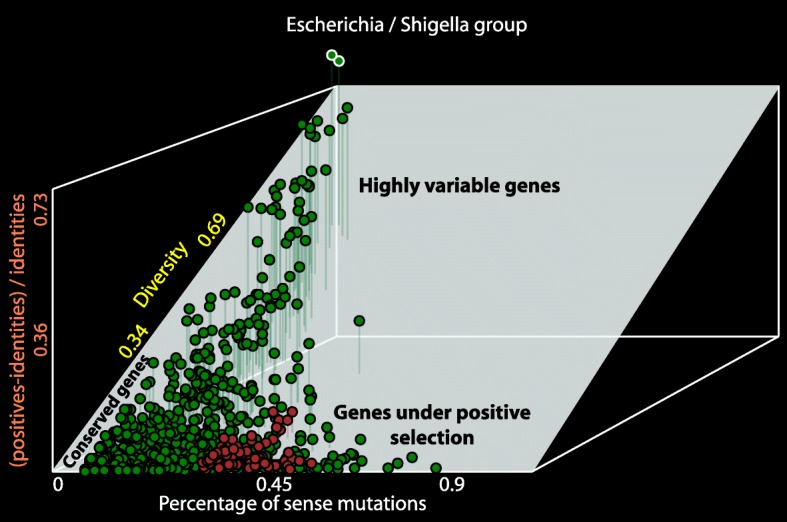
Graphical output of the Program BarcodeGenerator presents a distribution of COGs depicted by dots in the 3D plot. X-axis: percentage of sense mutations; Y-axis: 1 – percentage of identities; Z (vertical) axis: (positives-identities)/identities. Conserved, positively selected and highly variable groups of COGs are labelled. COGs suitable for barcoding are in brown colour

The principle aim of this project was the creation of a program, which for a given set of genomes would compare all pairs of genomes and select barcodes from the core genome. The workflow of the program is shown in Fig. 1. First the program identifies clusters of orthologous genes (COG) by means of reciprocal BLASP alignments with a cut-off e-value 0.0001. Then COGs are classified to the core genome and accessory parts of genomes. Core genes are constituent in all sampled genomes (core genome) and accessory genes are specific for one or several genomes (accessory genome). In the next step, MUSCLE codon alignment of the core COGs is performed [20]. Figure 2 shows a graphical output of the program BarcodeGenerator. COGs are depicted by dots projected into 3D space, where the X-axis is the percentage of sense mutations over the total number of nucleotide substitutions; the Y-axis is the difference between protein sequences (1 – percentage of identities); and the Z-axis (vertical axis) is the ratio (positives-identities)/identities. The COGs from the analysis can be grouped into several categories: conserved; positively selected; and highly variable genes. The conserved genes under moderate positive selection (highlighted in brown) proved to be suitable for barcoding [11]. Appropriateness of COGs for barcoding was scored as X × (1 ─ X) × (1 ─ Y) / (Z + 1), where X, Y and Z are values of the respective axes in Fig. 2. COGs are ordered by these scores from large to small and then nucleotide sequences of the genes from high scoring COGs are concatenated into barcode sequences until the requested length for barcodes is achieved. The order and locations of individual genes in barcode sequences are reported in text output files. Examples of output files for generated barcodes are accessible at http://seqword.bi.up.ac.za/barcoder_help_download/barcodes/ through the corresponding *info* hyperlinks.

**Fig. 3:**
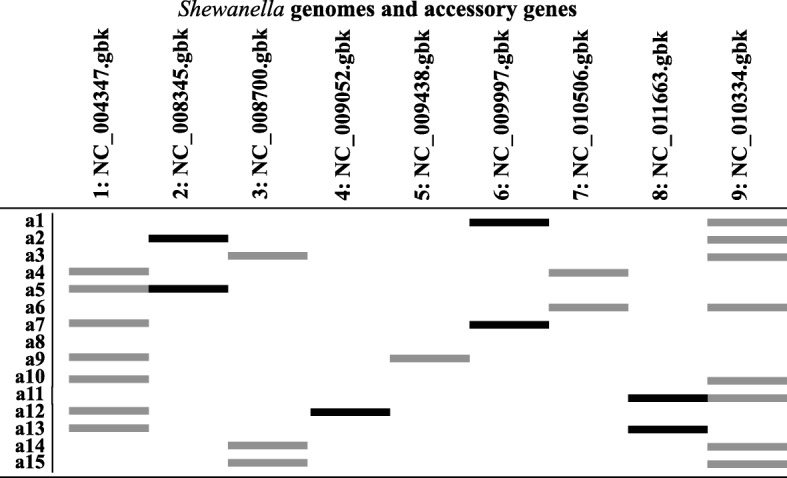
Distribution of 15 accessory genes (depicted by black and grey bars) selected to represent genetic variability of 9 sampled genomes of *Shewanella*

The program furthermore allows the addition of genes from the accessory genome to the barcodes. An example of accessory genes selected for 9 genomes of *Shewanella* is shown in Fig. 3.

To test the program, several sets of bacterial genomes representing different species of the genera *Lactobacillus*, *Mycobacterium* and *Shewanella*, and different strains of the group *Escherichia*/*Shigella* were used to create diagnostic barcodes. Analysis of functions of genes selected by the program BarcodeGenerator for diagnostic barcodes revealed that the most abundant group were comprised of genes encoding for ribosomal proteins. This finding is in agreement with many publications reporting ribosomal proteins as the most suitable taxonomic and phylogenetic markers used in rMLST [13, 14]. Ribosomal proteins comprised up to 15% of the sequences selected for barcodes by the program BarcodeGenerator. Other genes belonged to purine and pyrimidine biosynthetic pathways, ABC transporters, tRNA synthetases and amido-transferases, various oxidoreductases, acyl carrier proteins and several other functional categories. Among accessory genes, the most frequent were IS1 and IS2 transposases and orf2/orfB genes, *ynhF*-type membrane proteins, phage related transcriptional regulators and capsular polysaccharide biosynthesis proteins.

### Analysis and visualization of metagenomes by using barcode sequences

**Fig. 4:**
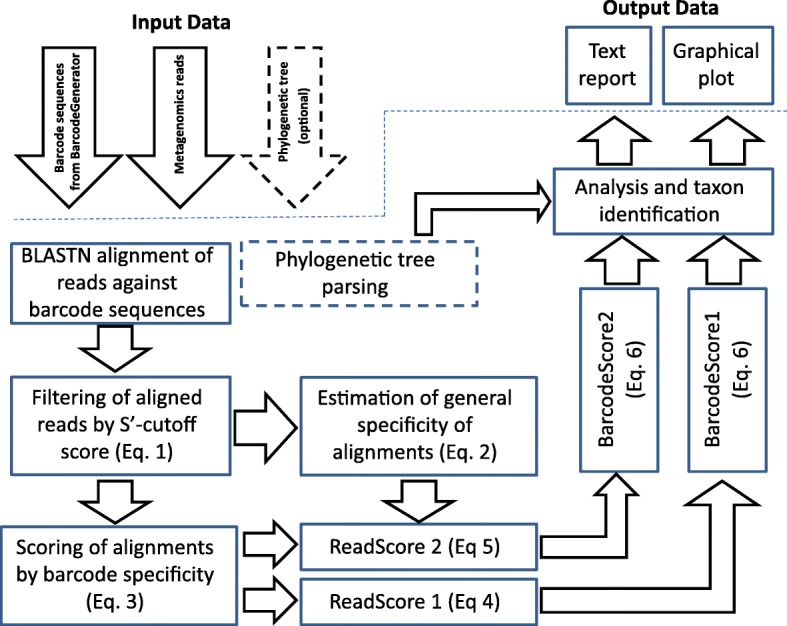
Workflow diagram of the program Barcoding 2.0

Of all the NGS technologies, Roche 454, Illumina and Ion Torrent systems are the mostly used for metagenomic samples [21, 22]. Recently, Roche 454 became obsolete and gave way to new technologies: PacBio, MinION and Oxford Nanopore. However, public databases still contain many metagenomic datasets generated by older technologies. Barcode sequences designed by BarcodeGenerator can be used for data mining in metagenomic sets of relatively short-reads generated by Roche 45, Illumina and Ion Torrent. This approach may not be applicable for the analysis of metagenomes generated by PacBio and Oxford Nanopore technologies due to high rate of sequencing errors and computational inefficacy of BLAST alignment of long-reads. Barcoding 2.0 is an application written in Python 2.7 (also compatible with Python 2.5) with a command-line user interface, available from the BarcodeGenerator website (http://bargene.bi.up.ac.za/). Workflow of the program is shown in Fig. 4. The program uses BLASTN to align reads against the generated barcode sequences and then calculates several parameters for scoring the results of the BLASTN alignment and individual barcodes. First, read alignments with BLASTN scores below an estimated *S′* score cut-off value are filtered out. The cut-off *S′* is calculated by the Eq. 1:

1 $$ {S}^{\prime }=S+\frac{L-S}{1+{e}^{\frac{3\left(L-S\right)}{S\times \mathit{\lg}(N)}}}-10\times \left(\mathit{\ln}\left(\frac{2S+100}{L+100}\right)-1\right) $$

where *S* – an average BLASTN score of all aligned reads; *L* – an average length of reads; and *N* – number of aligned reads.

The program then calculates the alignment specificity (*a*_*specificity*_) of read alignments (Eq. 2) by estimating the number of metagenomics reads (*N*_*aligned_reads*_), which were successfully aligned against the given number of barcodes sequences (*N*_*barcodes*_); and the total number of BLASTN matches (*N*_*matches*_):

2 $$ {a}_{specificity}=1-\frac{N_{matches}-{N}_{aligned\_ reads}}{N_{aligned\_ reads}\times \left({N}_{barcodes}-1\right)} $$

Values of specificity are in an interval from 0 to 1. The value of 0 indicates no specificity, i.e. every read in a given metagenome has found a match in every barcode sequence in the set. The value 1 means that every read matches specifically to one barcode sequence – maximal specificity.

Thereafter the program calculates the specificity of every read (*r*_*specificity*_):

3 $$ ReadScore1=\frac{BLASTN\_ score}{read\_ length}\times \frac{{\mathrm{r}}_{specificity}+ EXP\left({r}_{specpficity}\times {r}_{vicinity}\right)+1}{r_{specificity}+ EXP\left({r}_{vicinity}\right)+1} $$

It can be seen from eq. 3 that if one read is aligned against all barcodes, its specificity is 0; and if the read is aligned only against 1 barcode, its specificity is 1.

Then the program calculates two scores, ReadScore1 and ReadScore2 for every aligned read per barcode by Eqs. 4 and 5, respectively:

4 $$ ReadScore1=\frac{BLASTN\_ score}{read\_ length}\times \frac{{\mathrm{r}}_{specificity}+ EXP\left({r}_{specpficity}\times {r}_{vicinity}\right)+1}{r_{specificity}+ EXP\left({r}_{vicinity}\right)+1} $$

5 $$ ReadScore2={a}_{specificity}\frac{N_{read s\mid barcode}}{N_{read s}}\times \frac{BLASTN\_ score}{read\_ length}\times \frac{r_{specificity}+{1.5}^{\left({r}_{specificity}\times {r}_{vicinity}\right)}+1}{r_{specificity}+{1.5}^{\left({r}_{vicinity}\right)}+1} $$

It should be emphasized, that *ReadScore2* is barcode specific, i.e. reads aligned to several barcodes will have different *ReadScore2* values but the same value of *ReadScore1*. In Eqs. 4 and 5, the coefficient *r*_*vicinity*_ was calculated for every read to avoid downgrading of those reads, which were aligned to several barcodes of closely related organisms. First, a matrix of Jaccard distances is calculated for the set of barcodes, where the distance between two barcodes is 1 – *number_of_common_reads / total_number_of_reads*. If one read is aligned to several barcodes, the parameter *r*_*vicinity*_ for this read is calculated as 10 × *max_barcode_subset_distance / max_matrix_distance*. Values of *r*_*vicinity*_ are in the interval from 0 to 10. If the read is specifically aligned against only one barcode, its *r*_*vicinity*_ is 0. If the read is aligned against several barcodes of closely related organisms, the parameters *r*_*vicinity*_ will be small and the read will be scored high. However, if the read is promiscuously aligned against many unrelated barcodes, the parameters *r*_*vicinity*_ will be high and the read will be scored low.

After scoring all the aligned reads, the program calculates scores for every barcode to identify the corresponding species in the metagenome sample. Scores *BarcodeScore1* and *BarcodeScore2* (Eq. 6) are calculated from *ReadScore1* (Eq. 4) and *ReadScore2* (Eqs. 5) respectively. These scores are independent of the lengths of barcode sequences.

6 $$ {BarcodeScore}_i=\frac{1+{\sum}_i ReadScore}{1+\frac{3\times {BarcodeLength}_i\times \sum BLASTN\_ score}{4\times {\sum}_i^N BarcodeLength}}-1 $$

### Validation of the barcoding programs on artificial metagenomes

MetaSim is a sequencing simulator [19]. This program was used to generate collections of DNA reads from chosen bacterial genomes to design artificial metagenomic datasets with known species composition and species abundance. Metagenomes of different sample sizes (of 10,000, 50,000, 100,000, 300,000 and 500,000 reads) were generated by random selection of DNA fragments from the following genomes: *Shigella dysenteriae* Sd197 [NC_007606] – 15% of reads; *Escherichia coli* BL21 [NC_012947] – 10%; *E. coli* C ATCC 8739 [NC_010468] – 5%; *Lactobacillus fermentum* IFO 3956 [NC_010610] – 15%; *L. plantarum* [NC_004567] – 10%; *L. sanfranciscensis* TMW1 [NC_015978] – 5%; *Shewanella* sp. MR-4 [NC_008321] – 15%; *S. frigidimarina* NCIMB 400 [NC_008345] – 10%; *S. amazonensis* SB2B [NC_008700] – 5%; *Mycobacterium avium* 104 [NC_008595] – 5% and *M. abscessus* ATCC 19977 [NC_010397] – 5%. Artificial metagenomes generated for this work are available from http://seqword.bi.up.ac.za/barcoder_help_download/. Barcode sequences with lengths of 10, 25, 50, 75, 100, 150, 200 and 250 kbp were generated by the program BarcodeGenerator for the groups of genomes *Escherichia/Shigella*, *Lactobacillus, Mycobacteria* and *Shewanella*. Generated barcode sequences are available for download from the project website. In all these barcodes, sequences of core and accessory genes composed 70% and 30% of the total barcode sequence length, respectively. Lengths of the reads were normally distributed in a range from 200 to 350 bp.

Artificial metagenomes were used for validation of the program when applied to metagenomes of different sizes using barcodes of different lengths, and for calculation of appropriate cut-off values for species identification in metagenomic samples. The program returns two barcode scores, which are calculated by Eq. 6, based on different read alignment statistics (Eqs. 4 and 5).

**Fig. 5:**
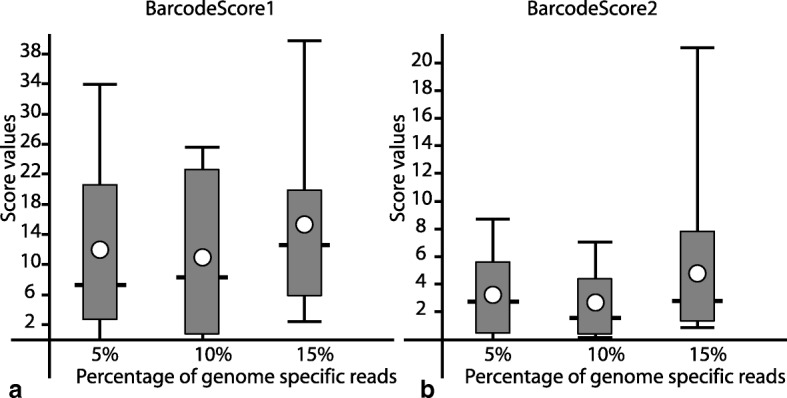
Distribution of values of **a** BarcodeScore1 and **b** BarcodeScore2 calculated based on the percentage of genome specific reads in artificial metagenomes. Whisker lines depict the minimal, maximal and median values; grey bars show middle quartiles and the open cycles indicate the average values

Values for *BarcodeScore1* and *BarcodeScore2*, which are dependent on the percentage of reads in a metagenome, are shown in Fig. 5a and b, respectively. *BarcodeScore1* is more sensitive to the presence of specific reads in metagenomes and is appropriate for a quantitative identification of taxa, while *BarcodeScore2* reflects better the abundance of specific reads in metagenomes.

Taxonomic units are identified in metagenomic samples by comparison of the calculated barcode scores to the precomputed cut-off values. True positives (TP) would be the genomes which were used for preparation of the artificial metagenomes and correctly identified by the program. Numbers of these genomes not identified by the program are false negatives (FN). False identification of other genomes represented in a set of barcodes leads to false positives (FP); but those excluded from the program output are true negatives (TN). To evaluate the barcoding performance with different cut-off values, the parameters of sensitivity, specificity and the ratio of true positives over false predictions TP / (FP + FN) were calculated.

**Fig. 6:**
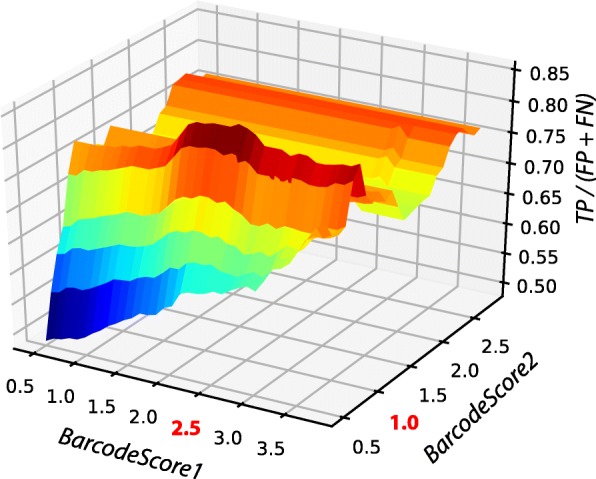
Surface plotting of the distribution of values for TP / (FP + FN) calculated for different pairs of cut-off values of the *BarcodeScore* 1 and 2

Distribution of values for TP / (FP + FN) calculated for combinations of *BarcodeScore1* and *BarcodeScore2* cut-offs is shown in Fig. 6.

The highest proportion of true positives over false predictions was achieved for the pair of cut-offs: *BarcodeScore1* = 2.5 and *BarcodeScore2* = 1.0. However, cut-off values *BarcodeScore1* = 2.3 and *BarcodeScore2* = 0.5 were set as the default to allow for higher sensitivity.

**Fig. 7:**
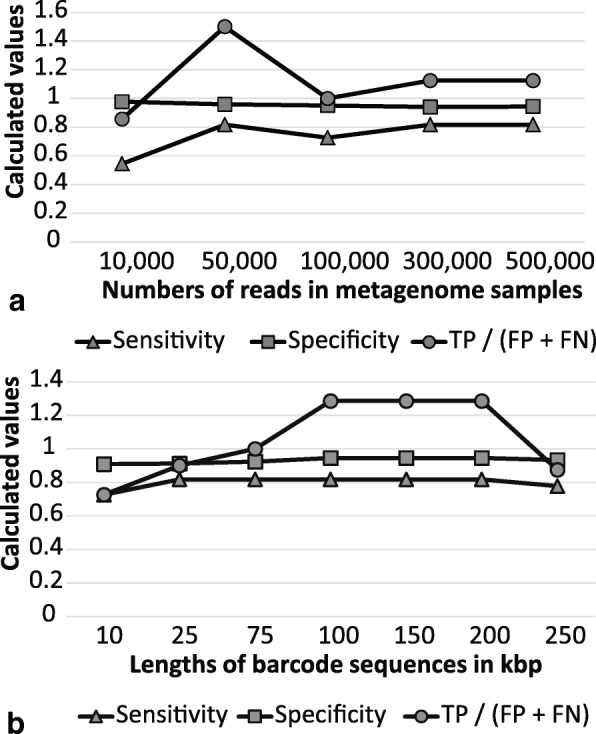
Influence of the **a** metagenome sample size and **b** length of barcode sequence on the program performance

The barcoding program with default cut-off values was used for processing of artificial metagenomes of different sample sizes using generated diagnostic barcodes of different lengths and different number of selected genes (all available from http://seqword.bi.up.ac.za/barcoder_help_download/barcodes/). It was found that the sample size (number of reads in a metagenome) had no effect on the sensitivity and specificity of the algorithm in the interval from 10,000 to 500,000 (Fig. 7a). In this range of values, the percentage of true positives increased with the sample size proportionally with the number of false positives. The ratio TP / (FP + FN) was higher in smaller metagenomes. In these experiments the metagenomic datasets of different sizes were aligned against barcodes of the same sequence length (50,000 bp).

Specificity and sensitivity was constant when using different lengths of barcode sequences (Fig. 7b). However, the ratio TP / (FP + FN) was optimal when the barcode sequences were in a range from 100 to 200 kbp. Shorter barcodes reduced the number of true positives as many reads remained unidentified and longer barcodes increased the number of false positive predictions. The influence of the barcode sequence length on the program performance was tested on artificial metagenomic datasets with 500,000 randomly generated reads.

**Fig. 8:**
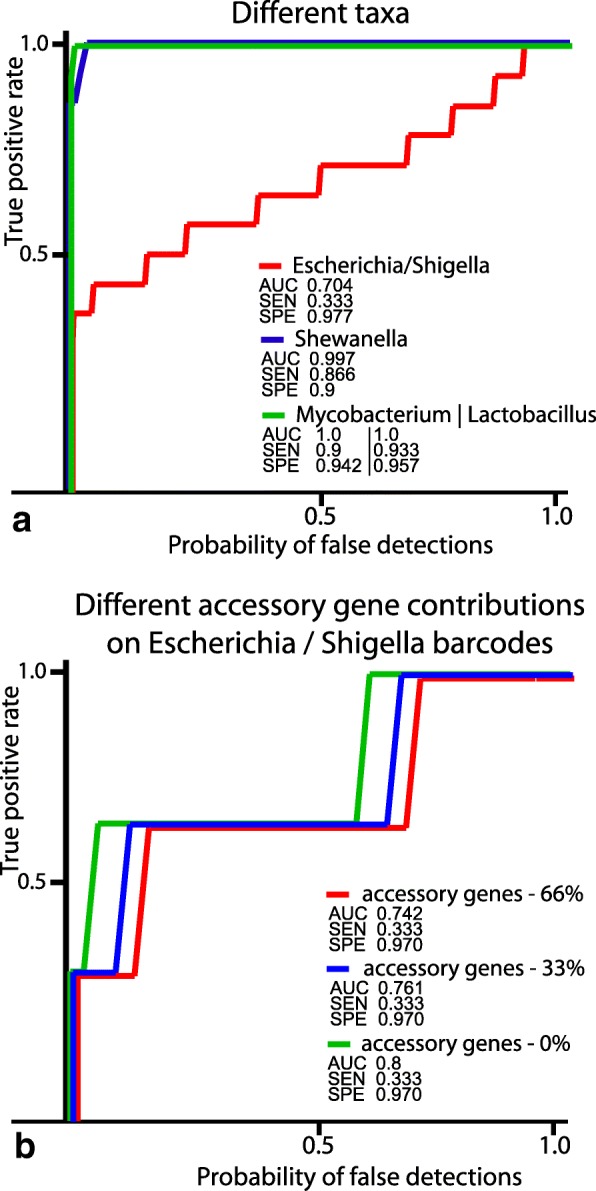
ROC diagrams of identification of **a** genomes on different taxonomic levels; **b** genomes of the *Escherichia / Shigella* group by barcodes with different contribution of accessory genes

Program performance was affected by the level of taxonomic relatedness between barcoded organisms. Receiver operating characteristics (ROC) curves were calculated for different taxonomic groups based on the results of identification of corresponding genomes in artificial metagenomic datasets (Fig. 8a). In addition to sensitivity and specificity parameters, the area under curve (AUC) was calculated, which is considered as a performance measure of diagnostic tools. Distinguishing between species of the same genus or family by the program was close to optimal. However, it was problematic for the program to differentiate between representatives of different clades of *Escherichia* and *Shigella*. It was assumed that including accessory genes in barcodes may improve the diagnostic performance. Comparison of identification results when the barcodes of the *Escherichia / Shigella* group of the same length (150,000 bp) with different proportions of core and accessory genes were used is shown in Fig. 8b. It was found that an increase in accessory genes in barcodes hampered distinguishing between closely related organisms compared with when the barcodes were based solely on core genes. This may be explained by the fact that related organisms frequently exchange mobile elements in a random fashion which impedes the proper differentiation between them. However, inclusion of species-specific accessory genes may improve the identification on higher taxonomic levels.

### BarcodeGenerator website and a case study of barcode guided species detection

**Fig. 9:**
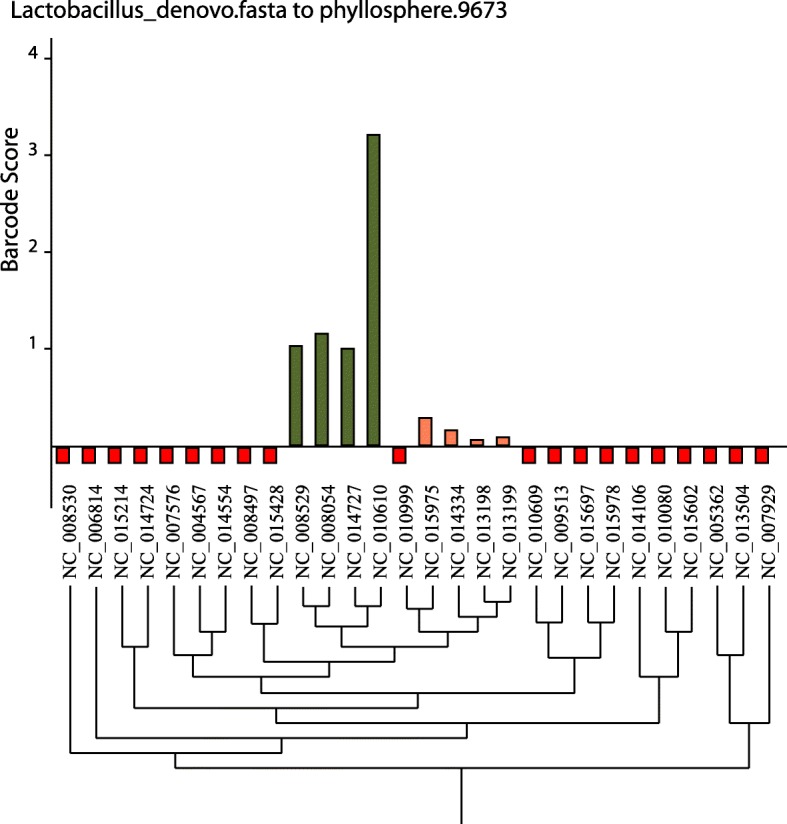
An example of identification of Lactobacillus species in phyllosphere metagenome

The program BarcoderGenerator is available at http://bargene.bi.up.ac.za/. This web application allows users to generate diagnostic barcodes based on genome sequences of species of interest submitted by users. Another program, Barcoding 2.0, with a command-line user interface is available for download from the Barcoder website. More details on the usage of these programs may be found on the help page http://seqword.bi.up.ac.za/barcoder_help_download/. Shortly, to generate a set of diagnostic barcodes, corresponding genome sequences in GenBank format should be uploaded to the server in a single archived file. Users can specify the length of barcode sequences and the required proportion of accessory genes in barcodes. The program will return a link to the output file with the generated barcode sequences in FASTA format, information on the genes selected for the barcodes and a graphical file in SVG format. An example of input and output files to test the program is available at http://seqword.bi.up.ac.za/barcoder_help_download/example/example.html. Generated barcodes may be used for binning metagenomics reads by using the command-line program Barcoding 2.0. The program performs a BLASTN alignment of reads against the barcode sequences and scores every barcode in the set as explained above (Fig. 4). The program returns the identification results in a text file and in a graphical SVG file. Results may be better visualized if the user provides the program with a phylogenetic tree file in Newick or Phylip format. An example of identification of *Lactobacillus* species by generated barcode sequences in the phyllosphere 9673 metagenome, publically available from MG-RAST database, is shown in Fig. 9. The program identified phylogenetically related strains *L. fermentum* (NC_010610) and *L. delbrueckii* (NC_008054, NC_008529 and NC_014727) depicted by green columns. The vertical axis shows estimated *BarcodeScore2* values, which reflects the relative abundance of identified organisms (see Fig. 5). The phylogenetic tree for the selected strains was created by a comparison of genome specific patterns of tetranucleotides with the program SWPhylo at http://swphylo.bi.up.ac.za/ [17]. The resulted tree file in PHYLIP format was provided through the command-line interface to the program Barcoding 2.0 as explained on the help Web-page http://seqword.bi.up.ac.za/barcoder_help_download/barcoding.html.

## Conclusions

In this paper a novel application, BarcodeGenerator (http://bargene.bi.up.ac.za), for the automatic generation of diagnostic barcode sequences was presented. BarcodeGenerator is an online tool for the selection of barcode sequences from a set of complete genomes provided by the users. It is easy to use and relatively fast. The program builds barcode sequences based on core genes of submitted complete genomes, but also allows addition of accessory genes to the barcodes. The output includes barcode sequences generated in FASTA format, information regarding the genes selected for the barcodes and a graphical file in SVG format.

In this study, barcode sequences were created for different groups of microorganisms (*Escherichia coli/Shigella*, *Lactobacillus*, *Mycobacteria* and *Shewanella*) to perform case studies. Ribosomal proteins, which have been reported by many publications as the most suitable genetic makers for taxonomic and phylogenetic studies, were the most abundant genes among selected marker genes.

Thereafter, another program was developed for binning of metagenomic reads against generated barcodes. The program uses BLASTN to align reads to the barcode sequences and then calculates scores for the BLASTN alignment and individual barcodes. After scoring all the aligned reads, the program calculates scores for every barcode to identify organisms present in metagenome samples. Taxonomic units are identified by comparison of calculated barcode scores to standard cut-off values set by default.

We also performed two experiments using varying metagenomes of different sample sizes and barcode sequences of different lengths. In the first experiment, metagenomic datasets of varying sizes (10,000 to 500,000 reads) were aligned against barcodes of the same length (50 kbp). We found that the sample size (the number of reads in a metagenome) had no effect on the sensitivity or specificity of the algorithm. In this range of values, the percentage of true positives increased with the sample size, proportionally to the number of false positives. The ratio of true positives over false predictions was higher in smaller metagenomes. Also, when varying lengths of barcode sequences (10 to 250 kbp) were aligned to a metagenomic dataset of 500,000 reads generated from randomly selected reads, the sensitivity and specificity also remained the same. However, the ratio TP / (FP + FN) was optimal when the barcode sequences were in the range from 100 to 200 kbp.

Receiver operating characteristic (ROC) curves of the algorithm performance were calculated for different microorganisms used in the artificial metagenomics datasets. Distinguishing between species of the same genus or family by the program was close to perfect but the program performed sub-optimally when distinguishing between strains of *Escherichia coli* and *Shigella*. Closely related organisms could be better identified when barcodes were based solely on core genes.

## Availability and requirements

**Project name:** Barcoder.

**Project home page:** http://bargene.bi.up.ac.za/.

**Operating system(s):** programs for download were tested on Linux and Windows.

**Programming language:** Python 2.7.

**Other requirements:** To run stand-alone program available for download from the project website, Python 2.7 has to be installed on PC.

**License:** no license;

**Any restrictions to use by non-academics:** no restrictions.

## Abbreviations

AUC: Area under curve
BLASTP: Basic local alignment search tool program
MLST: Multilocus sequence typing
NGS: Next generation sequencing
rMLST: Ribosomal multilocus sequence typing
ROC: Receiver operating characteristics
SEN: Sensitivity
SNP: Single nucleotide polymorphism
SPE: Specificity
wgMLST: Whole genome multilocus sequence typing
